# Increased Regulatory T Cells in Peripheral Blood of Acute Myeloid Leukemia Patients Rely on Tumor Necrosis Factor (TNF)-α–TNF Receptor-2 Pathway

**DOI:** 10.3389/fimmu.2018.01274

**Published:** 2018-06-05

**Authors:** Min Wang, Chen Zhang, Tian Tian, Teng Zhang, Ruiqing Wang, Fengjiao Han, Chaoqin Zhong, Mingqiang Hua, Daoxin Ma

**Affiliations:** ^1^Department of Hematology, Qilu Hospital of Shandong University, Jinan, China; ^2^Department of Hematology, Jinan Central Hospital, Affiliated to Shandong University, Jinan, China; ^3^Department of Pathology and Microbiology, University of Nebraska Medical Center, Omaha, NE, United States

**Keywords:** acute myeloid leukemia, tumor necrosis factor-α, regulatory T cells, tumor necrosis factor receptor-2, T helper cells

## Abstract

Acute myeloid leukemia (AML) harbors an immune suppression environment, featured by increased regulatory T cells (Tregs). The expression of tumor necrosis factor receptor-2 (TNFR2) on Tregs could be used to identify the maximally suppressive Treg population, and TNF-α furtherly promoted the expansion and function of Tregs *via* TNFR2 in mice. However, the role of TNF-α has not been determined in AML patients. In view of high levels of TNF-α and Tregs in AML patients, we hypothesized that the increased frequency of Tregs may rely on TNF-α–TNFR2 pathway. We investigated the levels of TNFR2^+^ Tregs and TNF-α secreted by T cells in peripheral blood (PB) of AML by flow cytometry and enzyme-linked immunosorbent assay, respectively. Our results showed the elevated plasma TNF-α in PB of newly diagnosed (ND) AML patients. The production of TNF-α by CD4^+^ T cells, especially by T helper (Th)17 cells was remarkably higher in ND AML patients than in complete remission (CR) patients and healthy controls. Then, we found that the circulating frequencies of CD4^+^CD25^+^ Tregs and CD4^+^CD25^high^ Tregs in AML patients were elevated compared with those in healthy controls and CR patients. TNFR2 expression was much higher on Tregs in AML patients and was preferentially expressed on CD4^+^CD25^high^ T cells. Furthermore, we confirmed that, *in vitro*, the additional TNF-α can increase the frequency of Tregs through TNFR2 in both AML patients and healthy controls. Summarily, in AML patients, the abnormally elevated level of TNF-α secreted by CD4^+^ T especially Th17 cells promoted the higher Tregs frequency *via* the TNF-α–TNFR2 pathway.

## Introduction

Acute myeloid leukemia (AML) is characterized by the proliferation of clonal neoplastic myeloid hematopoietic precursor cells and impaired production of normal hematopoiesis (1). It has been reported that immune system impairment exists in AML patients, and T cells as the most important part of immune system are found to be numerically and functionally defective. These defects are reported to influence the effect of regulatory T cells (Tregs), which suppress the proliferation and function of T helper (Th) cells (2, 3). Indeed, patients with AML show abnormally high level of Tregs within their peripheral blood (PB) and bone marrow (BM) compared with the healthy donors (4). Moreover, the presence of excessive Tregs correlates with the poor treatment outcome of AML patients (5). Therefore, understanding the mechanism and strategy of Tregs–Th regulating network in AML patients would broaden our horizons in the leukemia immunotherapy.

Over the past decades, numerous studies have been aimed to explore the mechanism of the abnormally increased Tregs. Grinberg-Bleyer et al. report that in mice, protection from diabetes by Tregs specifically relies on tumor necrosis factor α (TNF-α), a cytokine secreted by Th cells and traditionally considered as the proinflammatory factor (6). Chen et al. have first demonstrated that mouse peripheral Tregs express remarkably higher level of surface TNF receptor-2 (TNFR2) and TNF-α can promote the expansion and function of Tregs *via* TNFR2 (7). Furthermore, TNFR2 expression has recently been demonstrated to identify a subpopulation of Tregs with the maximally suppressive function in mice (8, 9) and other different diseases such as diabetes (10), malaria (11), and cancers (12). In a breast cancer mouse model, tumor-infiltrating Tregs are mainly composed of TNFR2^+^ Tregs (8). However, researches referring to TNFR2^+^ Tregs in AML have rarely been reported, and its related mechanism remains unclear.

In AML, leukemic blast cells directly promote the increase of Tregs frequencies through a variety of mechanisms, such as creating an immunosuppressive niche (13–16). However, the explicit underlying mechanism for the increased frequency of Tregs remains unknown. Therefore, we hypothesize that the abnormally elevated level of TNF-α would promote the higher frequency of Tregs through TNF-α–TNFR2 pathway in AML patients.

## Materials and Methods

### AML Patients and Healthy Donors

Forty-eight newly diagnosed (ND) AML patients (23 females and 25 males; age range, 21–83 years; median age, 45 years) and 34 complete remission (CR) AML patients (15 females and 19 males; age range, 19–71 years; median age, 40 years) were enrolled in this study. AML patients were diagnosed according to French–American–British classification system (17). CR was defined based on International Working Group Criteria (18). The control group consisted of 15 individuals (10 females and 5 males; age range, 18–67 years; median age, 39 years). Enrollment occurred between February 2013 and November 2014 in Qilu Hospital, Shandong University (Jinan, China). This study was approved by the Medical Ethical Committee of Qilu Hospital, Shandong University. The written informed consent was obtained from all patients before enrollment in the study in accordance with the Declaration of Helsinki.

### Enzyme-Linked Immunosorbent Assay (ELISA) for TNF-α

Peripheral blood samples were collected into heparin-anticoagulant vacutainer tubes. Plasma was obtained from all subjects by centrifugation and stored at −80°C for determination of cytokines. The level of TNF-α was determined with a quantitative sandwich ELISA in accordance with the manufacturer’s recommendations (lower detection limit 2.3 pg/ml; ELISA kits were from eBioscience).

### Flow Cytometric Analysis for Tregs Frequency and TNFR Expression by Tregs

Peripheral blood samples from all participants were collected into ethylenediamine tetraacetic acid-containing tubes. Peripheral blood mononuclear cells (PBMCs) were isolated by Ficoll-Hypaque (LiankeBio, China) gradient centrifugation, and then CD4^+^ T cells were isolated by human CD4 MicroBeads (Miltenyi Biotec, Germany) according to the manufacturer’s protocols. After blocking FcR, CD4^+^ T cells were incubated with appropriately diluted antibodies in the dark for 30 min. The antibodies used for surface staining were CD4-FITC, CD25-PerCP/Cy 5.5 (BioLegend, USA), TNFR1-PE, and TNFR2-APC (R&D systems, USA) antibodies. Isotype controls were utilized to enable correct compensation and to confirm antibody specificity. Stained cells were analyzed by flow cytometric analysis (BD science Pharmingen, San Jose, CA, USA).

### Flow Cytometric Analysis of TNF-α Expression by Th17 and Th1 Cells

Intracellular cytokines were studied by flow cytometry to reflect the percentages of corresponding cytokine-producing cells. Briefly, heparinized samples (100 μl) with an equal volume of Roswell Park Memorial Institute-1640 medium were incubated for 4 h at 37°C in 5% CO_2_ in the presence of 2.5 ng/ml of phorbol myristate acetate (PMA) (eBioscience), 1 μg/ml of ionomycin (eBioscience), and 1.7 μg/ml of monensin (BioLegend). PMA and ionomycin are pharmacologic T cell-activating agents that mimic signals generated by the T cell receptor complex and have the advantage of stimulating T cells of any antigen specificity. Monensin is used to block the intracellular transport mechanisms, thereby leading to an accumulation of cytokines in the cells. After incubation, 200 μl of sample was taken into flow tube and stained with CD4-PerCP/Cy 5.5 monoclonal antibody at room temperature in the dark for 20 min. After fixation and permeabilization, the cells were stained with IFN-γ-FITC, IL-17A-PE, and TNF-α-APC monoclonal antibodies. All antibodies were obtained from BioLegend. Isotype controls were utilized to enable correct compensation and to confirm antibody specificity. Stained cells were analyzed by flow cytometric analysis using an FACS calibur cytometer equipped with CellQuest software (BD science Pharmingen, San Jose, CA, USA). Th1 and Th17 cells were defined as CD4^+^IFN-γ^+^ and CD4^+^IL-17A^+^ T cells, respectively.

### The Effect of TNF-α/TNFR2 on the Frequency of CD4^+^CD25^+^Foxp3^+^ Tregs

The transcription factor forkhead box p3 (Foxp3) is a crucial intracellular marker and a key regulatory factor for the development and function of Tregs. To gain some appreciation of whether TNF-α can promote the increase of Tregs through TNFR2, we carried out the associated functional study. First, mononuclear cells were isolated by Ficoll-Hypaque gradient centrifugation. Mononuclear cells were incubated for 24 h in 24-well plate with IL-2 (100 U/ml) (Peprotech, USA) plus TNF-α (20 ng/ml) or IL-2 with TNF-α plus anti-TNFR2 antibody (R&D systems, USA) (1 μg/ml) while IL-2 alone as the negative control. Then the cells were collected and surface-stained with CD4-FITC (eBioscience) and CD25-APC (eBioscience) mouse monoclonal antibodies followed by incubating at room temperature in the dark for 20 min. Subsequently, the cells were fixed, permeabilized, and incubated with Foxp3-PE (BioLegend) monoclonal antibody for 1 h in the dark. The percentages of CD4^+^CD25^+^Foxp3^+^ Tregs were determined by flow cytometric analysis using an FACS calibur cytometer equipped with CellQuest software (BD science Pharmingen, San Jose, CA, USA). Isotype controls were utilized to enable correct compensation and to confirm antibody specificity.

### Statistical Analysis

Statistical significance was determined by performing unpaired *t* tests between healthy controls and AML samples. Paired *t* tests were performed when comparing the frequency of Tregs among IL-2 alone, IL-2 plus TNF-α, and IL-2 with TNF-α plus anti-TNFR2 antibody. Statistical analysis was performed using SPSS 15.0 (SPSS, Science, Chicago, IL, USA). *P* < 0.05 was considered statistically significant.

## Results

### Elevated Plasma Level of TNF-α in ND AML Patients

**Figure 1:**
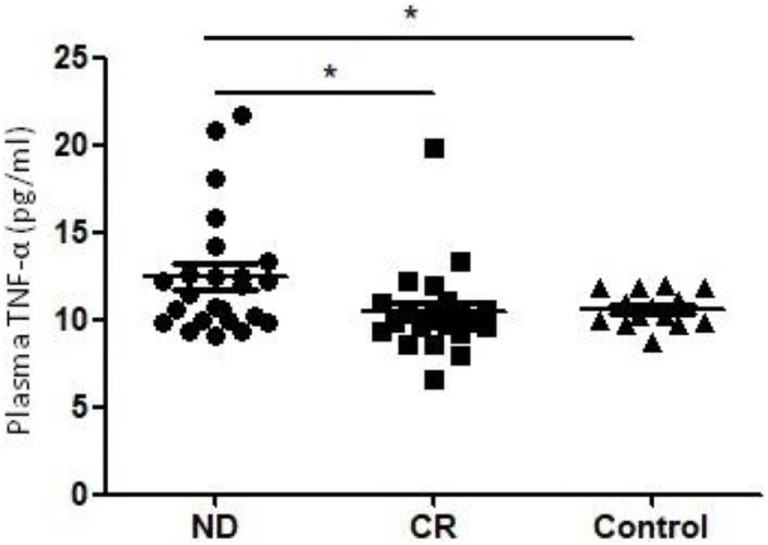
Plasma levels of tumor necrosis factor (TNF)-α in newly diagnosed (ND) acute myeloid leukemia (AML) patients, complete remission (CR) AML patients, and healthy controls. A significantly higher TNF-α level was observed in ND AML patients than in controls or CR patients (*P < 0.05).

The level of plasma TNF-α was significantly increased in ND AML patients (12.45 ± 3.48 pg/ml) compared with healthy controls (10.51 ± 2.49 pg/ml, *P* = 0.022). To further understand the influence of chemotherapy on the level of plasma TNF-α, we determined CR patients who was obtained after the standard induction chemotherapy. We observed a significant decrease of plasma TNF-α (10.61 ± 0.99 pg/ml, *P* = 0.028) in CR stage compared with in ND stage (Figure 1).

### Impaired Th1 and Elevated Th17 in AML Patients

**Figure 2:**
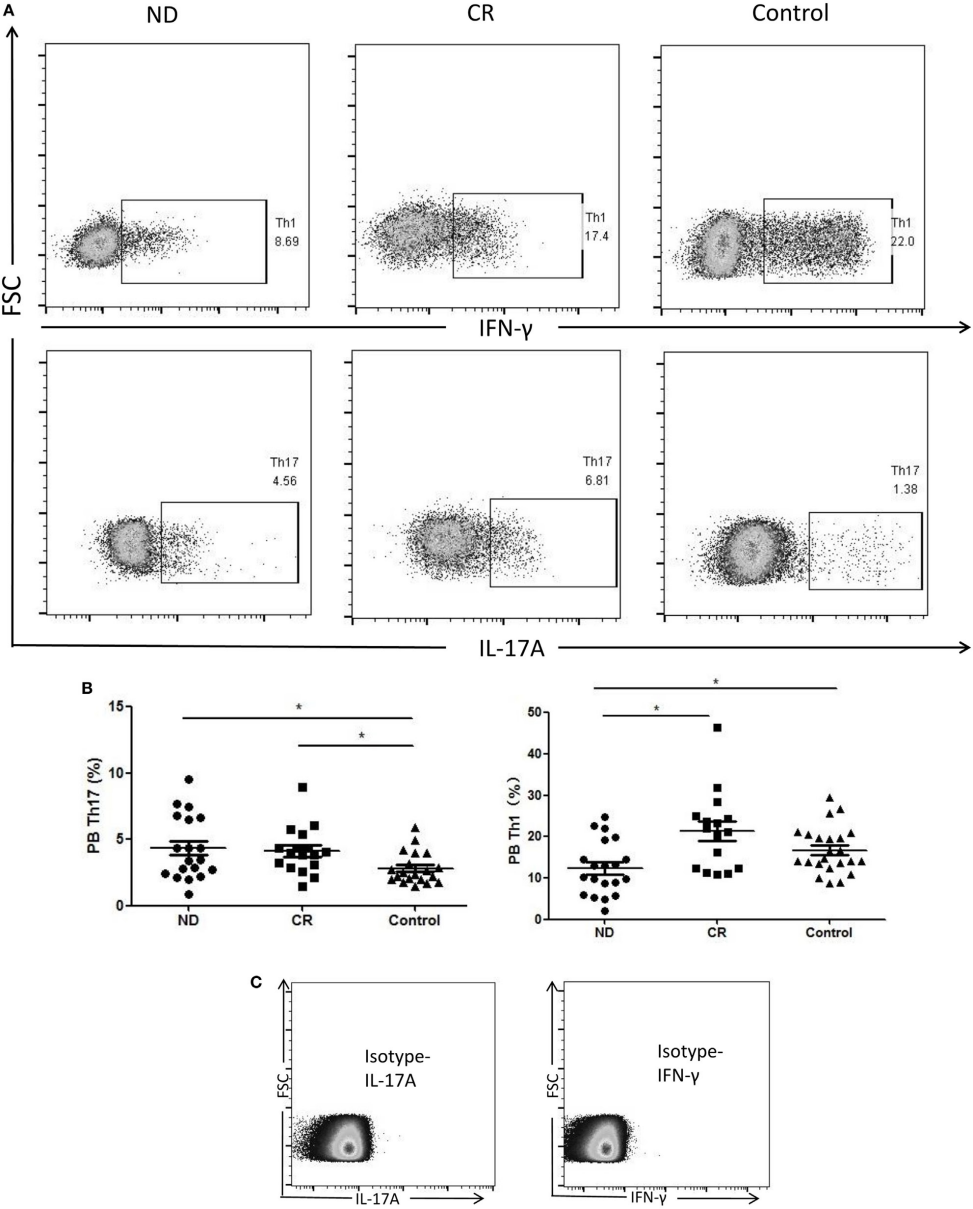
The results of circulating T helper (Th)1 and Th17 subsets in newly diagnosed (ND) acute myeloid leukemia (AML) patients, complete remission (CR) AML patients, and healthy controls. **(A)** Representative flow plots of Th1 and Th17 in ND AML patients, CR AML patients, and controls. **(B)** The percentage of circulating Th1 (CD4^+^IFN-γ^+^) cells was significantly decreased in ND AML patients compared with CR patients or controls; and Th17 (CD4^+^IL-17A^+^) cells was significantly increased in ND patients and CR patients compared with controls. **(C)** Isotypes were stained for IFN-γ and IL-17A.

We analyzed the circulating frequencies of Th1 and Th17 cells based on cytokine patterns after *in vitro* activation by PMA plus ionomycin and monensin in short-term culture. The circulating frequencies of Th1 cells were statistically decreased in ND AML patients (12.40 ± 6.50%) compared with the level of healthy controls (16.73 ± 5.61%, *P* = 0.024) or CR AML patients (21.30 ± 9.39%, *P* = 0.003). Moreover, we found that the circulating frequencies of Th17 cells were higher in ND AML patients (4.34 ± 2.39%, *P* = 0.016) or CR AML patients (4.13 ± 1.74%, *P* = 0.013), when compared with healthy controls (2.49 ± 1.24%). However, no statistical difference of Th17 level was found between ND and CR AML patients (Figure 2).

### Circulating CD4^+^ T Cells and Th17 Cells Produce Higher Level of TNF-α in ND AML Patients

**Figure 3:**
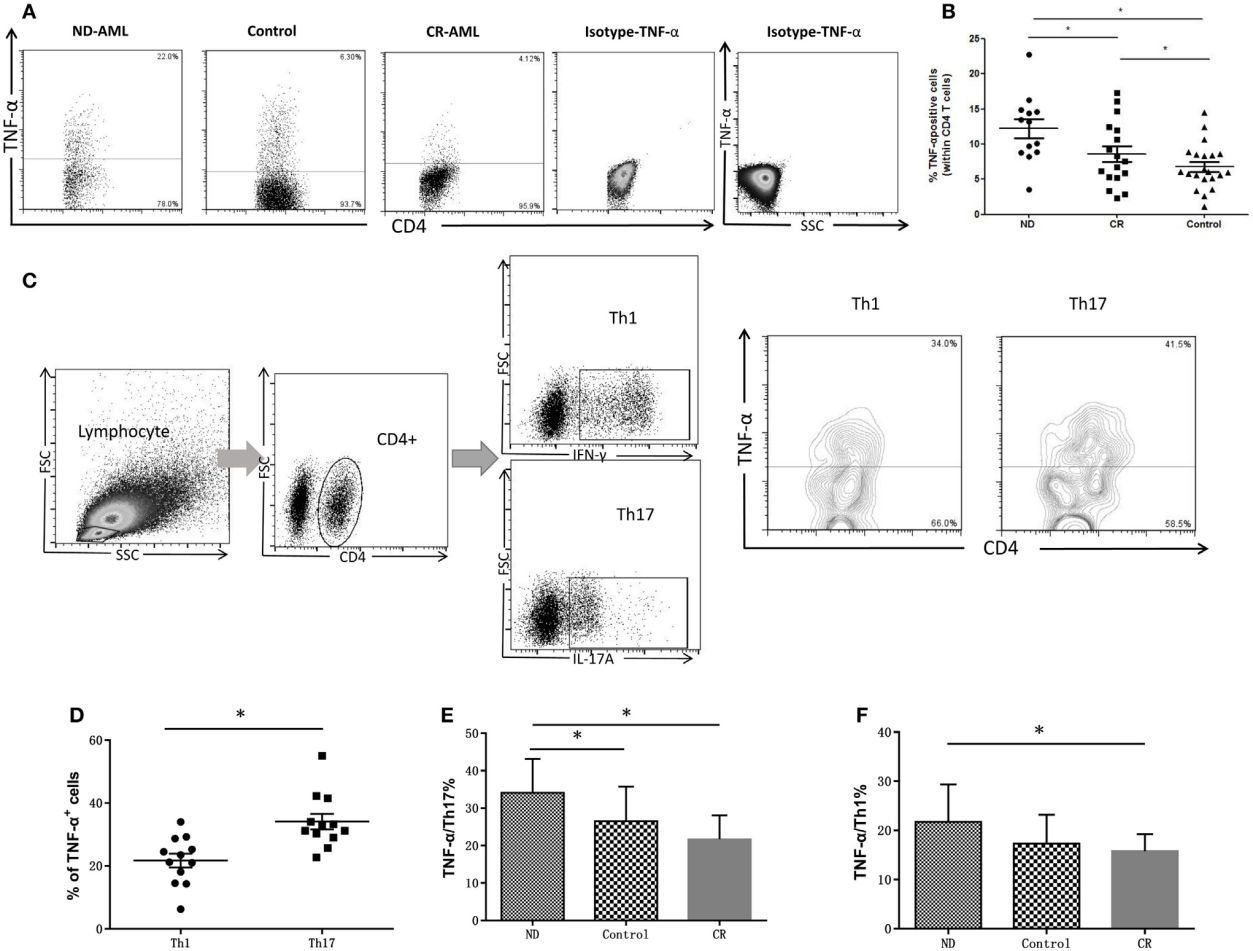
The intracellular tumor necrosis factor (TNF)-α expression in peripheral blood (PB) CD4^+^ T cells from acute myeloid leukemia (AML) patients and healthy controls. **(A)** Representative flow plots of intracellular TNF-α expression in PB CD4^+^ T cells from AML patients and healthy controls. Isotype for TNF-α was also performed. **(B)** The intracellular TNF-α expression was increased significantly in PB CD4^+^ T cells of newly diagnosed (ND) AML compared with complete remission (CR) patients or controls. **(C)** Representative flow plots of intracellular TNF-α expression by T helper (Th)17 and Th1 cells in PB of ND AML patients. **(D)** Intracellular TNF-α expression by Th17 cells was significantly higher than that of Th1 cells in PB of ND AML patients. **(E)** Intracellular TNF-α expression by Th17 cells in PB of ND AML patients was significantly higher than that of Th17 cells in PB of controls and CR patients. **(F)** Intracellular TNF-α expression by Th1 cells in PB of ND AML patients was significantly higher than that in controls or CR patients.

Cytoplasmic TNF-α level in circulating CD4^+^ T lymphocytes was analyzed by flow cytometry in AML patients and healthy controls. The production of TNF-α by CD4^+^ T cells from ND AML patients (12.21 ± 4.78%) was significantly higher as compared with that observed in healthy controls (6.78 ± 3.21%, *P* = 0.001) or CR AML patients (8.60 ± 4.62%, *P* = 0.046) (Figures 3A,B). As both Th1 and Th17 cells can produce TNF-α, we also determined the TNF-α-expression frequencies by Th1 or Th17 cells. We found that the frequencies of TNF-α^+^ cells in Th17 cells (34.04 ± 8.63%, *P* < 0.05) were significantly higher than those in Th1 cells (21.72 ± 5.09%) in ND AML patients (Figures 3C,D). Furthermore, Th17 cells in ND AML patients (34.11 ± 9.04%) maintained higher levels of TNF-α expression as compared with Th17 subsets in healthy controls (26.12 ± 8.62%, *P* < 0.05) and CR patients (21.68 ± 6.41%, *P* < 0.05) (Figure 3E). The percentage of Th1 in ND AML patients was also higher than that in CR patients (21.72 ± 2.21 vs 15.78 ± 3.48%, *P* < 0.05). However, no significant difference was found when compared the TNF-α production capacity of Th1 cells between ND patients and healthy controls (*P* = 0.079) (Figure 3F).

### Elevated Circulating Frequencies of CD4^+^CD25^+^ Tregs and CD4^+^CD25^high^ Tregs in Patients with AML

**Figure 4:**
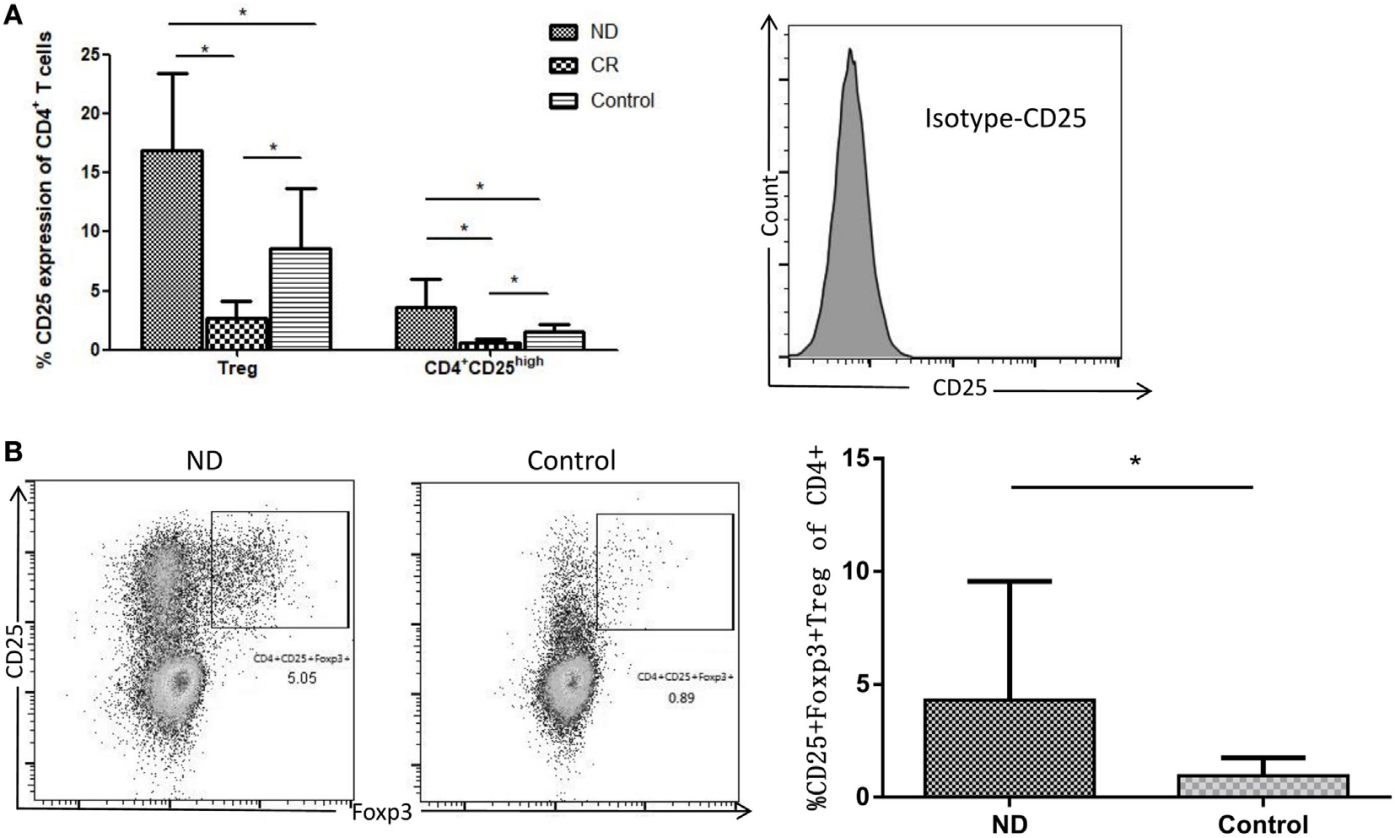
Comparison of CD4^+^CD25^+^ and CD4^+^CD25^high^ T cells in total CD4^+^ T cells from acute myeloid leukemia (AML) patients and healthy controls. **(A)** The frequency of CD4^+^CD25^high^ regulatory T cell (Treg) was determined as the percentage of brighter CD25^+^ cells on gated CD4^+^ T cells (based on forward and side scatter and CD4 staining). Isotype for CD25 was also performed. **(B)** CD4^+^CD25^+^Foxp3^+^ Tregs in the newly diagnosed (ND) AML patients were higher than that in controls. Representative flow plots of ND and controls were also performed.

Though CD4^+^CD25^+^ T cells were considered as Tregs, only cells expressing the highest levels of CD25 (termed CD25^high^) demonstrate potent regulatory function (19). Therefore, we determined the frequencies of CD4^+^CD25^+^ Tregs and CD4^+^CD25^high^ Tregs, respectively, in AML patients and controls. As shown in Figure 4A, the proportion of total CD4^+^CD25^+^ Tregs (16.93 ± 6.57%) or CD4^+^CD25^high^ Tregs (3.61 ± 2.36%) in the circulating CD4^+^ T cells of ND AML patients was markedly higher than that of healthy controls (8.61 ± 5.31%, *P* < 0.01; 1.48 ± 0.67%, *P* < 0.01; respectively) or CR patients (2.72 ± 1.43%, *P* < 0.01; 0.60 ± 0.31%, *P* < 0.01; respectively).

Forkhead box p3 is very important for the development and function of Tregs, therefore we determined the frequency of CD4^+^CD25^+^Foxp3^+^ Tregs. As shown in Figure 4B, CD4^+^CD25^+^Foxp3^+^ Tregs were also elevated in AML patients compared with controls (4.31 ± 1.41%; 0.97 ± 0.23%, *P* < 0.01; respectively).

### TNFR2 Expression on PB CD4^+^ T Cells, CD4^+^CD25^+^ Tregs, as Well as CD4^+^CD25^high^ Tregs Is Elevated in ND AML Patients

**Figure 5:**
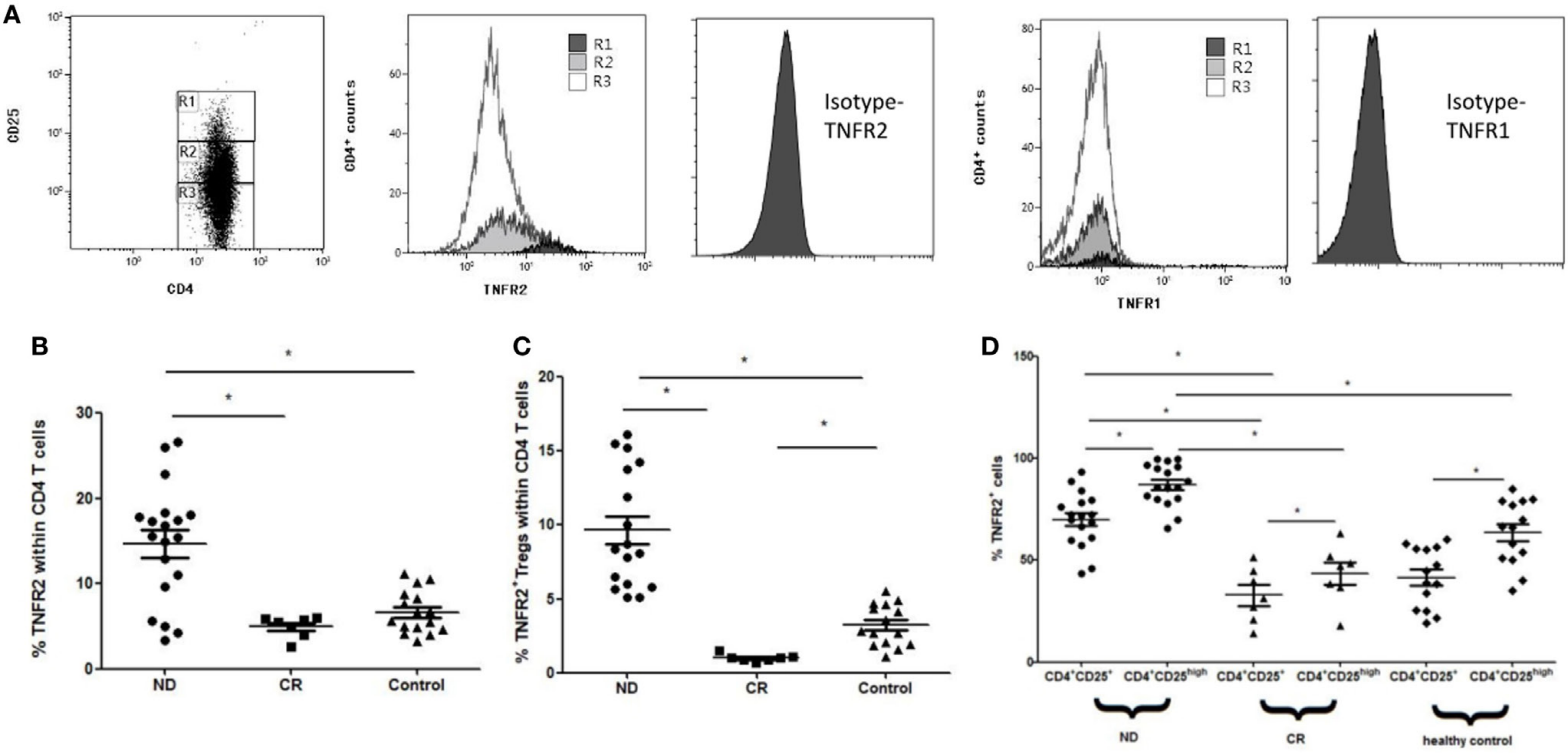
Levels of tumor necrosis factor receptor (TNFR)1 or TNFR2 expression on peripheral blood (PB) CD4^+^ T cells in acute myeloid leukemia (AML) patients and healthy controls. CD4^+^ T cells were stained for CD4, CD25, TNFR1, and TNFR2, and analyzed with FACS. **(A)** Representative flow plots of TNFR1 and TNFR2 expression by Rl (CD4^+^CD25^high^ T cells), R2 [CD4^+^CD25^+^ regulatory T cells (Tregs)], and R3 (CD4^+^CD25^−^ T cells) in PB of newly diagnosed (ND) AML patients. TNFR2 was preferentially expressed on CD4^+^CD25^high^ T cells, TNFR1 expression was unchanged. Isotypes for TNFR1 and TNFR2 were also performed. **(B)** The expression levels of TNFR2 on PB CD4^+^ T cells in AML patients and healthy controls. The TNFR2 expression on PB CD4^+^ T cells in ND AML was significantly higher than that in complete remission (CR) patients and controls. **(C)** The frequency of TNFR2^+^ Tregs in PB CD4^+^ T cells from ND AML patients was also significantly higher than that in healthy controls and CR patients. **(D)** The proportion of TNFR2^+^ cells by CD4^+^CD25^+^ Tregs and CD4^+^CD25^high^ T cells in AML patients and healthy controls. TNFR2 was preferentially expressed on CD4^+^CD25^high^ T cells compared with Tregs in PB of ND, CR patients, and controls. TNFR2 expressions on both PB CD4^+^CD25^+^ Tregs and CD4^+^CD25^high^ T cells in ND patients were significantly higher than those in CR patients and controls.

The percentage of TNFR2^+^ cells in PB CD4^+^ T cells from ND AML patients (14.67 ± 6.88%) was significantly higher than that from healthy controls (6.68 ± 2.51%, *P* < 0.01), and the level was decreased when AML patients reached CR (4.95 ± 1.24%, *P* < 0.01) (Figure 5B). Furthermore, the frequency of TNFR2^+^ Tregs in PB CD4^+^ T cells from ND AML patients (9.65 ± 3.99%) was also significantly higher than that in healthy controls (3.22 ± 1.4%, *P* < 0.01) and CR patients (1.01 ± 0.25%, *P* < 0.01) (Figure 5C). In our study, CD4^+^CD25^high^ T cells expressed higher proportion of TNFR2 when compared with CD4^+^CD25^+^ Tregs in healthy controls (63.27 ± 13.31 vs 41.48 ± 13%, *P* < 0.01) and ND AML patients (87.11 ± 3.35 vs 70.09 ± 16.57%, *P* < 0.01) as well as CR patients (43.45 ± 6.05 vs 32.81 ± 18.01%, *P* < 0.01) (Figure 5D). We also found that the expression of TNFR2 on PB CD4^+^CD25^+^ Tregs or CD4^+^CD25^high^ T cells in ND AML patients was markedly higher than that in healthy donors (*P* < 0.01) or CR AML patients (*P* < 0.01) (Figure 5D). By contrast, TNFR1 expression on CD4^+^ T cells remained unchanged regardless of CD25 expression levels (Figure 5A).

### TNF-α can Promote the Increase of Tregs Frequency Through TNFR2

**Figure 6:**
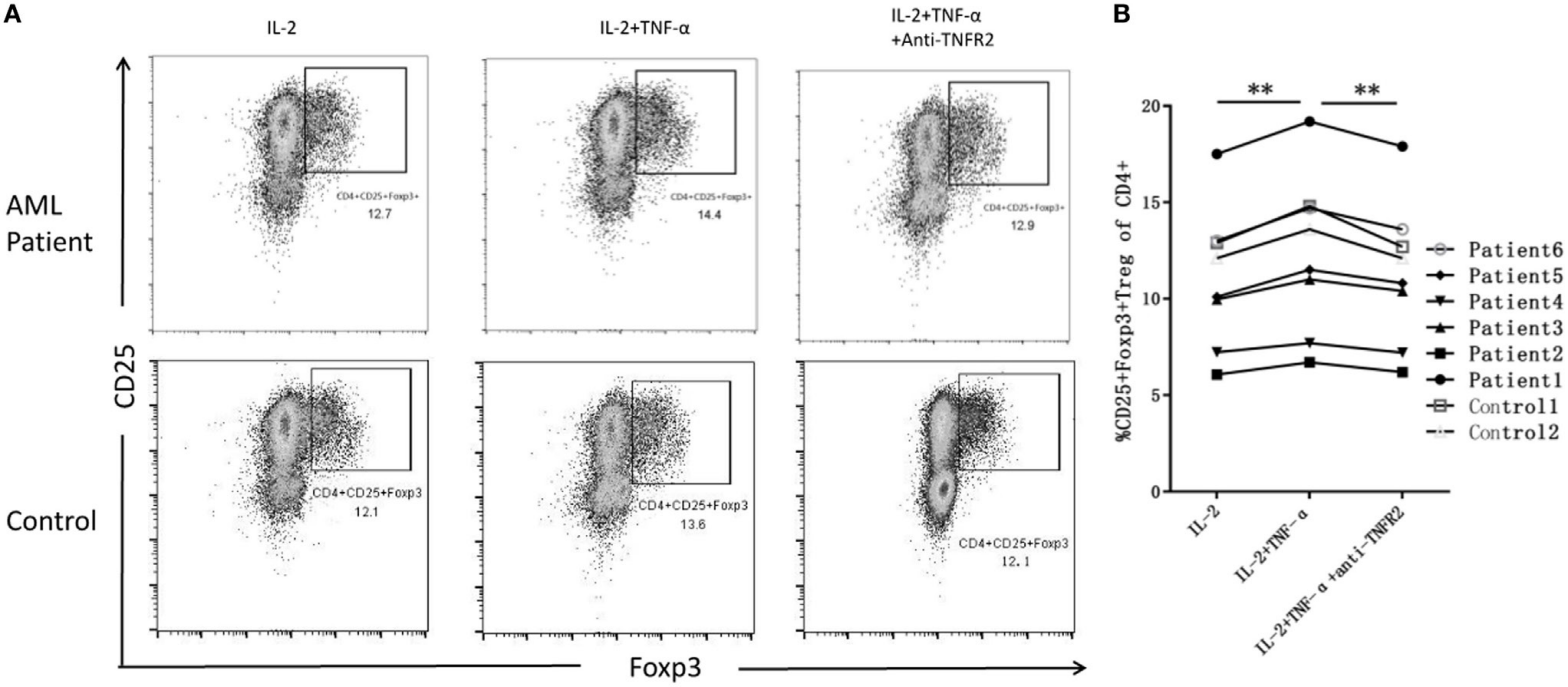
The expansion of regulatory T cells (Tregs) when treated with IL-2 alone, IL-2 plus tumor necrosis factor (TNF)-α, and IL-2 with TNF-α plus anti-TNF receptor-2 (TNFR2) antibody. **(A)** Representative flow plots of acute myeloid leukemia (AML) patients and controls confirm greater intracellular induction of CD4^+^CD25^+^Foxp3^+^ Tregs after added TNF-α, and the expansion of Tregs decreased when added anti-TIMFR2 antibody. **(B)** The expression of Tregs has been upregulated after added IL-2 plus TNF-α, and decreased when added anti-TNFR2 antibody in AML patients.

IL-2 is a protein that regulates the activities of leukocytes and is commonly used to maintain the survival of Th cells. We treat PBMCs from AML patients with IL-2 alone *in vitro*, and the frequency of Tregs is 10.64 ± 4.14%. Next, we use IL-2 plus TNF-α, the relative percentage increases to 11.80 ± 4.62%, and then we treat PBMCs with IL-2 with TNF-α plus anti-TNFR2 antibody, the frequency of Tregs decreases to 11.02 ± 4.30%. In our study, co-incubation of TNF-α and IL-2 can produce a significant increase in Tregs over IL-2 alone in ND AML patients (*P* < 0.01), and the usage of anti-TNFR2 antibody would eliminate such effect. We also stimulated PBMCs from two healthy donors by IL-2 and TNF-α, and no significant difference was found compared with PBMCs from AML patients (Figure 6).

## Discussion

Acute myeloid leukemia is an aggressive hematological malignancy represented by the accumulation of BM blast and hematopoietic failure. It was believed that various factors contribute to the AML tumorigenesis such as mutations (19, 20), radiation (21), and carcinogens (22). Th cells immunity has been shown to be dysregulated in the pathogenesis of AML, and the possible mechanisms have been identified as alterations in Th cells, such as apoptosis, abnormal secretion, or expression of cytokines, and signaling molecules (23). Although the disorders of Th cells immunity in the pathogenesis of AML have long been recognized, the explicit mechanism remains unknown. Accumulating evidences indicate that the imbalance between Th cells and Tregs plays a pathological role in AML pathophysiology, and it can be the potential target for further researches and future therapies (24, 25).

Increased frequencies of Tregs have been proved in AML patients. Moreover, Tregs from AML patients mediate vigorous suppressive activity compared with those from healthy controls, and it is correlated with the poor clinical prognosis (4, 5). Recent studies have reported that TNFR2^+^ Tregs represent a highly potent Treg subset (26). Previous studies showed that TNF-α–TNFR2 interaction is critical for the activation and expansion of functional Tregs (7). Until now, several studies about highly inhibitory TNFR2^+^ Tregs in human tumor microenvironment have demonstrated that the elevated level of TNFR2^+^ Tregs represents a subset with stronger suppressive capacity. However, there is rare data about TNF-α–TNFR2 pathway in AML.

In the present study, we confirm that the levels of circulating Tregs are higher in ND AML patients as compared with healthy controls or CR patients. Interestingly, compared with healthy controls, the expression levels of TNFR2 on PB CD4^+^ T cells, CD4^+^CD25^+^ Tregs, as well as CD4^+^CD25^high^ T cells are considerably increased in ND AML patients, and then the expression levels of TNFR2 decreased markedly when patients achieved remission. Furthermore, TNFR2 is preferentially expressed on CD4^+^CD25^high^ T cells compared with CD4^+^CD25^+^ Tregs in AML patients or in healthy controls. These results are similar with a previous study that TNFR2^+^ Tregs proportions reduced within PB of all patients after the induction therapy (27, 28). Our data indicate that the frequency of TNFR2 expression on PB CD4^+^ T cells could become a novel and easily accessible marker, which can be used to predict clinical outcomes or monitor the progress of AML patients subjected to standard cytotoxic therapy.

Tumor necrosis factor-α has been found to play an important role in promoting the development and progression of malignant diseases. Targeting TNF-α with TNF antagonists has elicited an objective response to certain solid tumors in phase I and II clinical trials. Previous reports have demonstrated that TNF-α is involved in the pathogenesis of AML, and in the progress of leukemogenesis, including cellular transformation, proliferation, and angiogenesis. Previous studies reported that serum TNF-α level was significantly higher in AML and, more importantly, the high serum TNF-α level is an adverse prognostic factor for survival and event-free survival in AML patients (29). Our data also revealed that in AML patients, plasma TNF-α levels are upregulated and decreased markedly within CR patients. However, we showed comparable levels of plasma TNF-α but significant decreased CD4^+^CD25^+^ as well as CD4^+^CD25^high^ Tregs in CR AML patients compared with healthy controls. The inconsistency may be because that plasma TNF-α can also be produced by other cells besides Tregs.

Although elevated TNF-α has shown correlated with poorer prognosis in AML, the study about intracellular TNF-α in AML remains unclear. Mononuclear cells have been considered as the main sources of TNF-α production for a long time (30, 31). We confirmed that TNF-α can also be produced by T cells and retroact on T cells. Manipulating TNF-α activity has the possibility of tipping the balance between pathogenic Th cells and Tregs (32). Grinberg-Bleyer et al. stated that TNF-α produced by CD4^+^ T cells could act on Tregs to promote their expansion *via* TNFR2 in autoimmune diabetes (6). Thus, in our study, we demonstrate that CD4^+^ T cells produce more TNF-α in AML than in healthy controls. The production of TNF-α by PB CD4^+^ T cells in ND AML patients was significantly higher than that in healthy controls. When patients achieved CR, the TNF-α production decreased obviously. Furthermore, we find that Th17 cells expressed a significantly higher level of TNF-α compared with Th1 cells in ND AML patients, and the capacity of Th17 cells to produce TNF-α is much higher in ND patients than in healthy controls.

Previous studies revealed that the quantitative or functional imbalance of Th17 cells and Tregs has been involved in the occurrence and development of AML. However, the interplay between Th17 cells and Tregs in AML is still not quite clear. A recent evidence indicates that TNF-α plays a vital role in the regulation of Th17 cells and Tregs in some inflammatory and autoimmune diseases and tumors (33). Our results clarify that Th17 might play an important role in the overproduction of TNF-α and TNFR2 had been shown much higher in Tregs in ND AML patients. Furthermore, TNF-α may bind to the highly expressed receptor, which then increase the frequency of Tregs.

In our study, we demonstrate the direct evidence that TNFR2^+^ Tregs that is presented at high levels in AML patients could be used as a prognostic biomarker for chemotherapy. Elevated Th17 frequencies as well as enhanced ability of secreting TNF-α contribute to the overproduced TNF-α. Moreover, our data provides a novel insight about the complex interplay between Th17 and Treg subsets in which Th17 cells potentially stimulate the increased frequency of Tregs. This effect is mainly mediated by TNF-α–TNFR2 signaling pathway.

## Ethics Statement

This study was carried out in accordance with the recommendations of Guidelines for the Care and Use of Human Sample, Medical Ethical Committee of Qilu Hospital, Shandong University. The protocol was approved by the Medical Ethical Committee of Qilu Hospital, Shandong University. All subjects gave written informed consent in accordance with the Declaration of Helsinki.

## Author Contributions

Conceived and designed the experiments: DM and MW. Performed the experiments: MW and CZ. Analyzed the data: TZ, RW and FH. Contributed reagents/materials/analysis tools: TT, CqZ, and MH. Wrote the paper: DM, MW, and CZ.

## Conflict of Interest Statement

The authors declare that the research was conducted in the absence of any commercial or financial relationships that could be construed as a potential conflict of interest. The reviewer DW and handling Editor declared their shared affiliation.
